# Land use shapes riverine nutrient and sediment concentrations on Moorea, French Polynesia

**DOI:** 10.1038/s41598-025-13425-1

**Published:** 2025-07-31

**Authors:** Kyle Neumann, Christian John, Terava Atger, Tauira Punu, Jordan A. Hollarsmith, Deron E. Burkepile

**Affiliations:** 1 Marine Science Institute, University of California, Santa Barbara, Santa Barbara, CA 93106 USA; 2 Te Pu Atitia Center, University of California Gump Research Station, Moorea, French Polynesia; 3 Alaska Fisheries Science Center, NOAA Fisheries, 17109 Pt Lena Loop Road, Juneau, AK 99801 USA; 4 Department of Ecology, Evolution, & Marine Biology, University of California, Santa Barbara, Santa Barbara, CA 93106 USA

**Keywords:** Global change, Hydrology, Land use, Precipitation, River chemistry, Biogeochemistry, Freshwater ecology, Hydrology, Environmental chemistry, Environmental impact

## Abstract

Human activities drive changes in freshwater ecosystems by altering biogeochemical cycles. Freshwater networks provide important ecosystem services to human societies by purifying water and serving as an intermediary between terrestrial and marine systems. On high volcanic tropical islands, human activities are compartmentalized by steep terrain that delineates watershed boundaries. Patterns of land use affect adjacent stream ecosystems through runoff of sediment and nutrients, which fluctuates in the tropics as a result of seasonal rainfall. Here, we sought to reveal human impacts on nutrient and sediment regimes of tropical rivers by tracking patterns of river chemistry across a series of watersheds on Moorea, French Polynesia, between 2018 and 2019. Repeated sampling of rivers across a gradient of human activities revealed that water chemistry varied seasonally and with respect to rainfall and land use. In particular, dissolved inorganic nitrogen was more concentrated in rivers of watersheds with higher amounts of land clearing during the rainy season, and total suspended solids and phosphate were higher when recent rainfall was high. Importantly, the water quality of the rivers on Moorea repeatedly exceeded safe water quality standards established for similar high tropical islands in the Pacific. Our results show that differential land use across the landscape can have a substantial impact on the amounts of nutrients and sediment that tropical rivers transport, which on tropical islands could facilitate movement of materials from land to sea as precipitation increases with intensifying climate change.

**Supplementary Information:**

The online version contains supplementary material available at 10.1038/s41598-025-13425-1.

## Introduction

Human activity has altered approximately 75% of the Earth’s surface in the last millennium^1^. Global land-use models indicate a 0.8 million km^2^ loss in forest cover since 1960 and a corresponding increase of 0.9-1.0 million km^2^ in agricultural land^1^. Hydrologic models estimate that ~ 50% of observed increases in river run-off globally between 1900 and 2000 were the result of changes in land use as deforestation reduced water retention capacity of landscapes. This makes land-use change at least as impactful as climatic change for altering runoff patterns^2^. In addition to increases in water transport, human-driven land use change has led to an increase in nutrient enrichment and sediment loading in rivers^3–6^. Rivers and streams provide valuable ecosystem services in tropical fluvial networks, but these benefits are highly sensitive to disturbance^7^. For example, rivers’ capacity for self-purification is often negatively impacted by land use via increases in pollution and on alterations to planktonic diversity and abundance^8^. These negative impacts on key ecosystem services can be mitigated by maintaining forest cover and other riparian management strategies, emphasizing the importance of local decision-making and land management practices for preserving nature’s contributions to people^9^.

Deforestation in mountainous tropical regions can have an outsized impact on riverine sediment loading and discharge due to their steep slopes, highly erodible soils, and high precipitation^10,11^. The removal of forest vegetation exposes soil to erosion from precipitation and wind while also reducing the soil holding capacity of the cleared area by reducing vegetation root depth and root mass^12^. Elevated sediment concentrations in rivers increase turbidity and reduce light availability and can alter the geomorphology of riverbeds^13^. One study of Kolombangara, a high tropical island in the Solomon Islands, estimated that if no land management practices were put in place, a 10% increase in logging activity would result in an increase in sediment runoff of approximately 1500% island-wide, equating to an annual transport of approximately 3,000 tons of sediment from land through rivers to the ocean^14^. If forests are replaced by agriculture or population centers in these regions, nutrient loading in rivers would likely increase substantially. Conversion of native forest to agriculture utilizing high-nitrogen fertilizer could result in a 25-fold increase in nitrogen export from the system, and urbanization at the same scale could result in a nearly 50-fold increase in nitrogen export as compared to native forest^12^. Understanding the role of land use in nutrient and sediment runoff is essential to understanding alterations in aquatic ecosystems as well as effective planning, management, and restoration of altered watersheds.

Land use change has resulted in as much as a 20-fold increase in nitrogen (N) and phosphorus (P) concentrations of many rivers worldwide as compared to pre-industrial levels^12^. N and P enter aquatic ecosystems as a result of atmospheric deposition as well as excess fertilizer application on agricultural plots, industrial and domestic activities such as detergent use, and untreated or incompletely treated human and animal waste^12,15,16^. As either N or P tend to be the limiting nutrient for primary production in freshwater systems, elevated concentrations of these nutrients can have a number of deleterious effects including: the proliferation of algae and cyanobacteria resulting in increased levels of harmful algal toxins, reduction in dissolved oxygen, and alterations to the biodiversity of flora and fauna^13,17,18^. In addition to altering the absolute levels of N and P, anthropogenic nutrient loading in freshwater systems can alter the stoichiometric ratios of dissolved N and P, which can shift trophic interactions and biogeochemical cycling in freshwater ecosystems^18^.

Watersheds of high volcanic islands are particularly vulnerable to impacts of land clearing. In tropical areas, these islands often have high annual rainfall and periodic large storms that can quickly displace fertilizer from agricultural fields and increase riverine sediment loads^19–21^. In addition to the potential ecological impacts of runoff, people living on these islands are often dependent on rivers for clean water for drinking and bathing. Thus, freshwater bodies with increased nutrient and sediment pollution can reduce human health and well-being^22–24^ through exposure to runoff as well as algal and bacterial blooms. Tropical islands are experiencing rapid growth in their populations, tourism, industry, logging, and large-scale agriculture resulting in the clearing of forest for farms and developments^19,25,26^. As a result, it is important to understand the impacts of active development on the nutrient and sediment concentrations in rivers on tropical islands. Water quality standards for nutrient and sediment concentrations have been established for some tropical regions^[e.g.27^, but have not been established or adopted universally across the tropics. Thresholds for water quality standards are generally based on impacts of river chemistry for both human health^19^ and ecosystem function^28 ^so regularly exceeding water quality thresholds poses broader concerns for human and environmental outcomes.

**Fig. 1:**
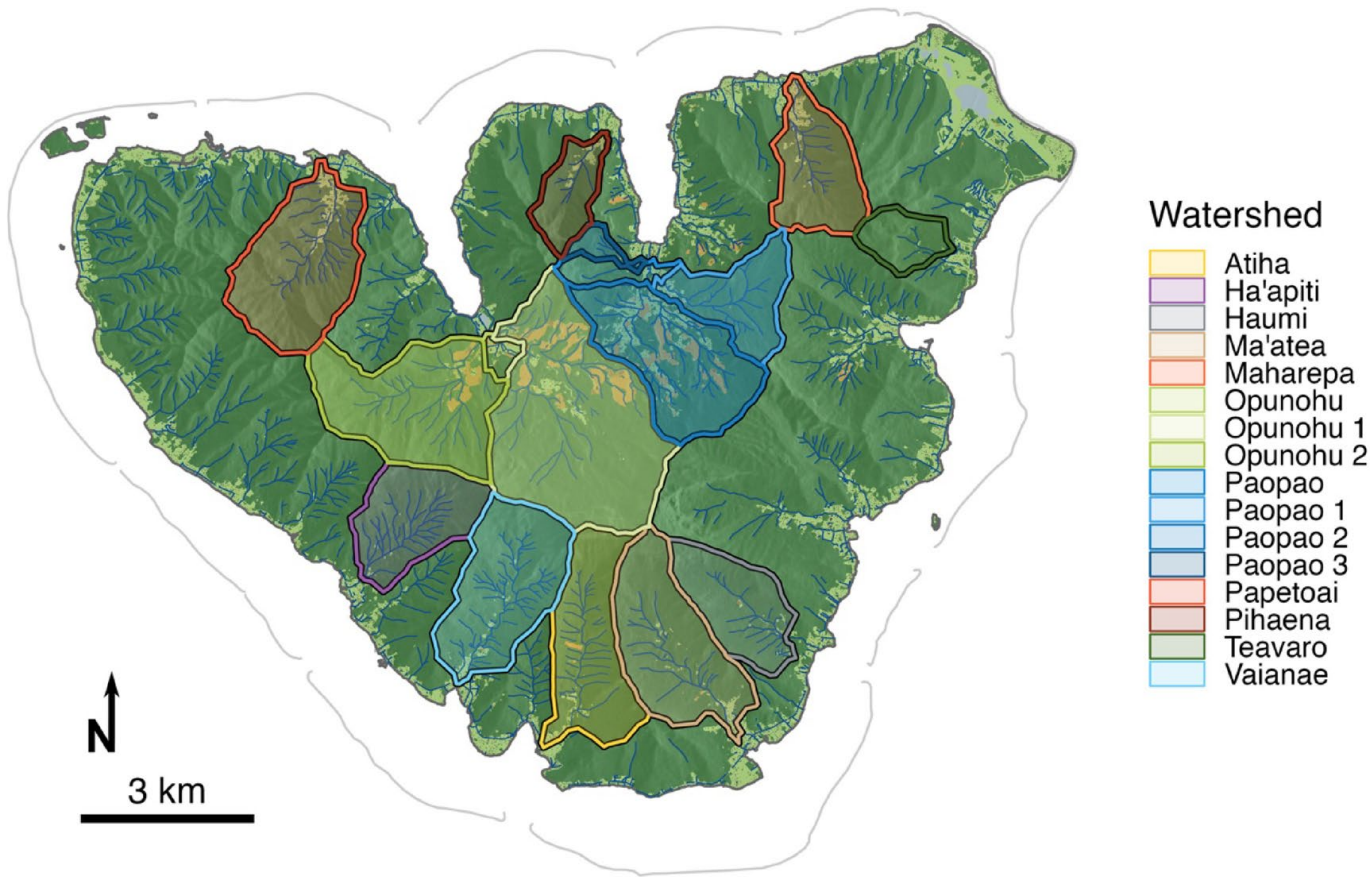
Moorea, French Polynesia with stream channels and focal watersheds superimposed on hillshaded land cover (forested areas shown in dark green, water in blue, intensive agriculture in orange, and other cleared land in pale green; see methods for classification details).

Here, we focus on rivers of Moorea, French Polynesia (Fig. 1), to examine how nutrient and sediment concentrations in surface waters relate to human alterations of the landscape. Moorea is a high volcanic island with a rapidly changing landscape due to increased tourism, housing, and industrial development as well as increasing agriculture^29,30^. Many people on Moorea have raised issues regarding the state of rivers on the island, citing observations of turbid water, algal and bacterial blooms that result in rashes, and reduced abundance of a freshwater shrimp species that is an important traditional food (*TA and TP pers. obs.*). Further, Moorea is home to near-shore coral reef ecosystems, and both resident observations and recent academic work suggests that terrestrially-derived nutrients have a negative impact on near-shore reefs^30,31 ^which could compromise the ecosystem services (e.g. fisheries and tourism) they provide. However, to date no study has assessed nutrient and sediment loading in rivers on Moorea as it relates to land use. In response to this community and scientific need, we tested the following hypotheses about watersheds of Moorea:

1. Rivers in watersheds with higher proportions of cleared land will have higher sediment loading.
2. Rivers in watersheds with higher proportions of cleared land and higher populations will have high concentrations of N and P.
3. Water nutrient and sediment concentrations will increase following recent precipitation.
4. Rivers in watersheds with the highest percentages of land use and higher populations will exceed nutrient and sediment thresholds established for human and environmental protection in similar systems.

To test these hypotheses, we collected water samples from 16 streams on Moorea across rainy and dry seasons in two years. Water samples were analyzed for nitrate (NO_3_^−^), nitrite (NO_2_^−^), and ammonium (NH_4_^+^), the three N species that comprise dissolved inorganic nitrogen (DIN), as well as orthophosphate (PO_4_^3−^) and total suspended solids (TSS). We integrated stream chemistry data with precipitation, watershed size, land use, and census data, and used principal component analysis (PCA) and linear modeling to evaluate how local factors influenced the nutrient and sediment dynamics of streams across the island. Finally, we compared nutrient and sediment loading in streams of Moorea to similar systems in other tropical islands using previously-established water quality thresholds to assess potential concerns regarding human health and alteration to ecosystem function.

## Results

### Patterns in rainfall

**Fig. 2:**
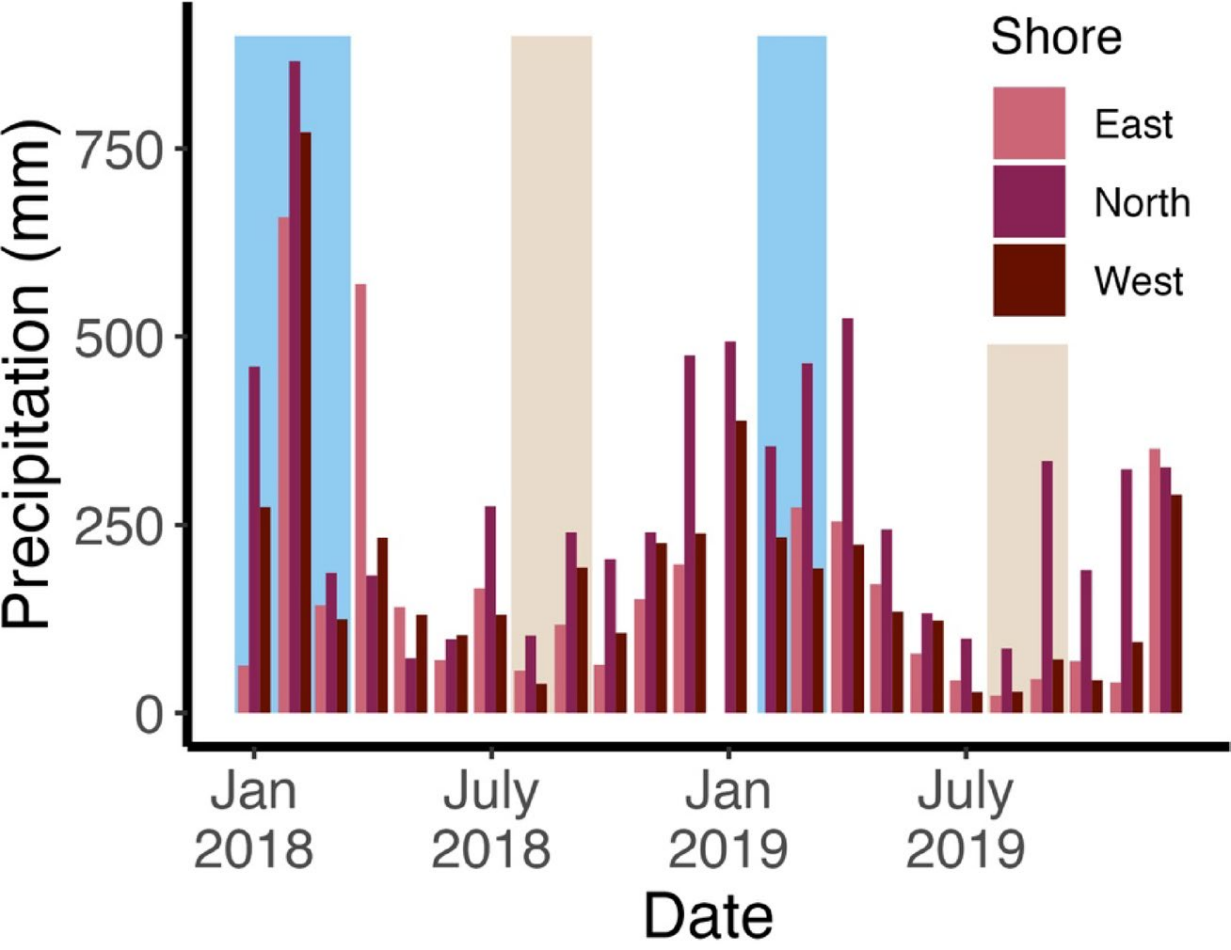
Monthly rainfall by shore orientation (2018–2019). Rainy season sampling was in January – March 2018 and February – March 2019 (blue background). Dry season sampling occurred during August – September in both 2018 and 2019 (beige background). No rainfall data were reported at the east shore meteorological station (Afareaitu 2) during January and February, 2019.

Precipitation in Moorea was seasonal but occurred during every month of the study period (Fig. 2). Within our sampling dates, February of 2018 had the highest cumulative monthly rainfall with 866 mm of precipitation on the North shore, 659 mm on the East shore, and 772 mm on the West shore. The rainy season in 2019 had lower monthly precipitation than in 2018 with a peak of 234 mm in February on the West shore, and 465 and 274 mm in March on the North shore and East shore, respectively. August was the driest month of both years and precipitation totaled 103 mm on the North shore, 56 mm on the East shore, and 39 mm on the West shore in 2018; and 86 mm of on the North shore, 23 mm on the East shore, and 28 mm on the West shore in 2019.

### Watershed and seasonal differences in water chemistry

**Fig. 3:**
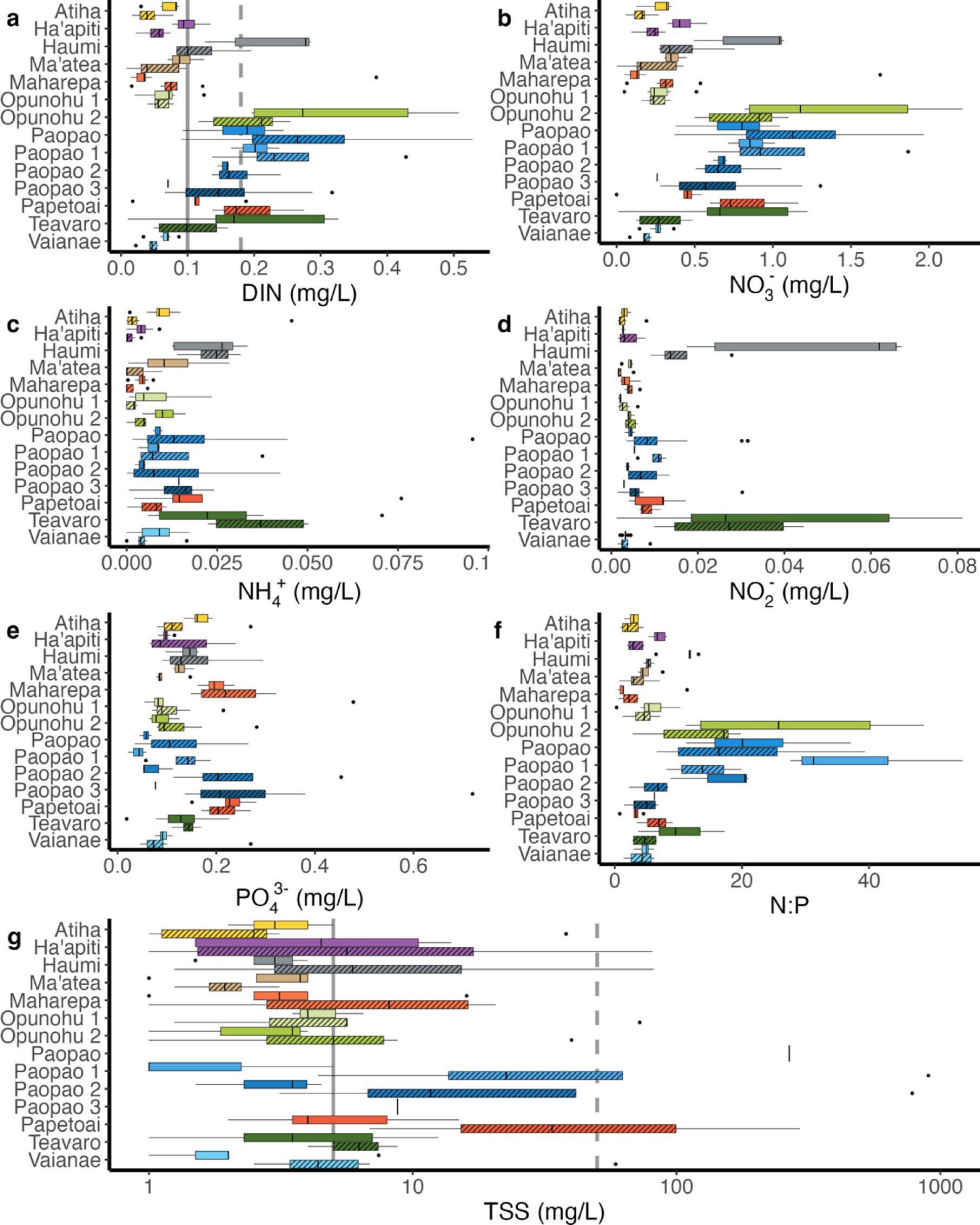
Nitrogen concentrations as dissolved inorganic nitrogen (DIN,** a**), nitrate (NO_3_^−^,** b**), ammonium (NH_4_^+^,** c**), nitrite (NO_2_^−^,** d**), phosphate (PO_4_^3−^,** e**), Nitrogen:Phosphorus ratio (N:P,** f**), and total suspended solids (TSS,** g**; note log scale on x axis) across sites and seasons (dry season indicated by an open box and rainy season as a striped box). Boxes indicate the first and third quartiles, the vertical bar shows the median, and the vertical lines extend to the upper and lower 1.5 times the interquartile range. Outliers are shown as dots. In (**a**) and (**g**), water quality thresholds are shown as vertical grey lines in background, with the more relaxed water quality threshold as a dashed line and the more stringent threshold as a solid line.

Water chemistry varied considerably by nutrient, season, and watershed across the sampling period (Fig. 3, Supplementary materials S1). DIN concentrations ranged from a minimum of 0.008 mg/L measured in a sample collected from Ma’atea in 2019, to 0.528 mg/L measured in a sample collected in Paopao in 2018. DIN differed significantly across watersheds (*p* < 0.001) and seasons (*p* = 0.04; Fig. 3a, Supplementary materials S2). We found no significant differences between seasons within each watershed in the post-hoc Tukey test, though the significant interaction between watershed and season in the overall model indicates that rainy season DIN concentrations were higher than dry season in some watersheds (e.g., Papetoai and Maharepa) while the opposite pattern was observed in others (e.g., Ha’apiti and Haumi). The watershed with the highest mean ± s.e. DIN concentration was Paopao (0.256 ± 0.018 mg/L), and the lowest mean concentration was found in Vaianae (0.057 ± 0.004 mg/L).

The N species comprising the majority of DIN was NO_3_^−^, followed by NH_4_^+^ and finally NO_2_^−^. NO_3_^−^ concentrations differed significantly by watershed (*p* < 0.001) and season (*p* = 0.02), and there was a significant interaction between watershed and season (*p* < 0.001); like DIN, the post-hoc Tukey test revealed no significant differences in NO_3_^−^ concentrations between seasons within watersheds (Fig. 3b). NH_4_^+^ concentrations differed significantly by watershed (*p* < 0.001), while the interaction with season was not significant. Teavaro and Haumi had higher NH_4_^+^ levels than the other watersheds (Fig. 3c; the mean ± s.e. NH_4_^+^ concentration in these watersheds was 0.030 ± 0.006 mg/L and 0.023 ± 0.003 mg/L, respectively, whereas across watersheds the mean NH_4_^+^ concentration was 0.012 ± 0.002 mg/L). NO_2_^−^ concentrations differed significantly by watershed (*p* < 0.001) but not by season although there was a significant interaction of watershed and season (*p* = 0.001). The rainy season NO_2_^−^ concentrations were significantly lower than the dry season concentrations in Haumi (difference ± s.e. = 0.030 ± 0.011 mg/L, *p* < 0.001). Like NH_4_^+^, Teavaro and Haumi had higher NO_2_^−^ values relative to the other watersheds (Fig. 3d; NO_2_^−^ concentration in these watersheds was 0.035 ± 0.007 mg/L and 0.033 ± 0.008 mg/L, respectively, whereas across watersheds the mean NH_4_^+^ concentration was 0.009 ± 0.003 mg/L).

PO_4_^3−^ concentrations ranged from a minimum of 0.018 mg/L from a sample collected in Teavaro in the dry season to a maximum of 0.72 mg/L in a sample collected from Paopao 3 in the rainy season. PO_4_^3−^ differed significantly among watersheds (*p* < 0.001) and season (*p* = 0.03) and had a nearly significant interaction of watershed and season (*p* = 0.053) (Fig. 3e). The highest mean PO_4_^3−^ concentration from rivers that flowed year-round was observed in Paopao 3 (0.257 ± 0.053 mg/L) while the lowest was in Paopao 1 (0.093 ± 0.024 mg/L). PO_4_^3−^ concentrations were significantly associated with TSS concentrations (β = 0.04, *p* < 0.001, and r^2^ = 0.33). The N:P ratio ranged between a minimum of 0.29 in Opunohu 1, and a maximum of 54.7 in Paopao 1. The mean N:P ratio was highest in Paopao 1 (24.2 ± 5.97) and lowest in Maharepa (2.29 ± 0.64). N:P differed significantly across watersheds (*p* < 0.001) and had a significant interaction with watershed and season (*p* < 0.001), with Paopao 1 and Opunohu 2 having significantly higher N:P molar ratio in dry seasons than the rainy seasons (Fig. 3f).

TSS concentrations ranged from below the detection limit in some samples from Atiha, Ha’apiti, and Maharepa to a maximum of 902 mg/L found in a sample collected in Paopao 1 during the rainy season. Mean TSS was higher during the rainy season than the dry season in 10 of the 12 watersheds for which TSS data were collected in both rainy and dry seasons (i.e. all sites except Pihaena, Paopao, and Paopao 3). Differences in TSS among seasons were significant (*p* = 0.002) but the interaction between watershed and season was not significant (*p* > 0.05) (Fig. 3). Although differences in TSS among watersheds were not significant after accounting for season, variability in TSS concentration across watersheds was impressive: the lowest mean TSS concentration was recorded in Ma’atea at 2.5 ± 0.44 mg/L while the highest was recorded in Paopao 1 at 137 ± 128 mg/L. The next highest mean TSS concentration was in Paopao 2 at 117 ± 111 mg/L, followed by Papetoai with 46 ± 36 mg/L, less than half the concentration of the two largest Paopao sub-watersheds.

### Land-use and human population across Moorea

Our analysis of land use focused on the percent of cleared land in each focal watershed using the consensus land cover classification from a series of Worldview-3 images collected in 2018 (Fig. 1)^32^. Island-wide model accuracy was 98% based on a spatially random sample of validation points. Using a hierarchical sampling scheme to balance across training classes, classification accuracy was 82%. Most of the classification discrepancies came from errors of omission of exposed soil, which was often co-occurring with intensive monoculture and captured by that class, and errors of omission of small buildings which were lost as noise to their surrounding vegetation buffer. In both cases, these discrepancies fell within the broader category of “cleared land” and so were determined to be acceptable. The watershed with the highest proportion of cleared land in 2018 was Paopao 2 (23%), followed by Opunohu 1 (10.4%) while the lowest was Ha’apiti (0.8%) (Table 1). The human population on Moorea was 17,463 residents in 2017, with the largest population in the Paopao basin (1281 residents) and the smallest in Opunohu 2 (10 residents) (Table 1). Population was not significantly related to watershed size or percentage of the watershed that was classified as cleared land (*p* > 0.05 but β > 0 in all cases), but these null associations were driven by the large area and broad-scale land clearing in the Opunohu basin (which has a very low population density): among all watersheds except those in the Opunohu basin, population was positively associated with watershed size and percentage of watershed that was cleared (R^2^ = 0.60 and 0.32, and *p* = 0.002 and 0.04, respectively).

**Table 1:** Watershed parameters including orientation of shore, watershed area, population size, Strahler stream order of the sampled river, stream length from source to sampling location, proportion of the watershed that is clear of forest (including agriculture and development). Sub-watersheds comprising main Opunohu and Paopao basins indicated with italics.

Watershed	Shore	Watershed area (km^2^)	Stream order	Stream length (km)	Population	Cleared (%)
Atiha	W	4.3	3	5	239	3.4
Ha’apiti	W	3.3	3	3.1	372	0.8
Haumi	E	2.9	2	3.4	422	2
Ma’atea	E	4.8	3	4.9	518	3.5
Maharepa	N	3	2	3.5	698	8.8
Opunohu	N	15.2	4	5.4	159	9.5
*Opunohu 1*	N	9.2	4	5.2	149	10.4
*Opunohu 2*	N	5.6	3	4.2	10	5.7
Paopao	N	8.8	4	4.5	1281	15.6
*Paopao 1*	N	2.5	4	3.9	280	3.5
*Paopao 2*	N	5	3	3.3	721	22.7
*Paopao 3*	N	0.4	2	1.9	280	9.2
Papetoai	N	5	3	4.5	798	5.3
Pihaena	N	1.6	2	2.8	44	7.4
Teavaro	E	1.3	2	2	367	1.3
Vaianae	W	4.7	3	4.1	233	3

### Associations between precipitation, watershed development, and river chemistry

**Fig. 4:**
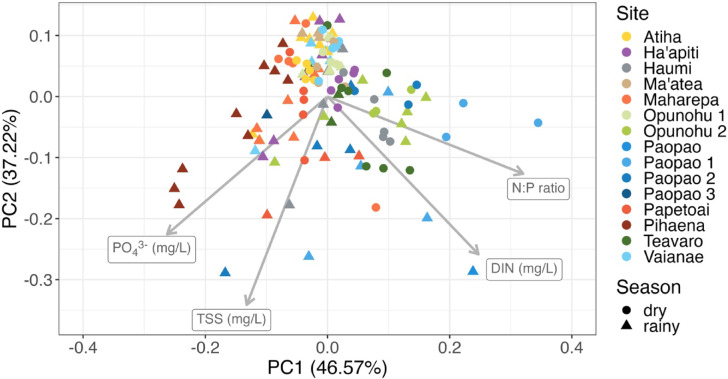
Principal component analysis (PCA) results of stream chemistry (DIN, PO_4_^3−^, TSS, and N:P) across watersheds. Stream chemistry loadings are shown in grey and scaled by 0.5. On the first PC axis, DIN and N:P loaded positively, while TSS and PO_4_ loaded negatively. On the second PC axis, all four elements loaded negatively. Each point represents one sampling event.

Sample points across the watersheds and seasons were distributed across the ordination space in the PCA (Fig. 4). The first PC axis explained 46.6% of the variability in river chemistry, and was negatively associated with PO_4_^3−^ and TSS (PC1 loadings of -0.53 and -0.26, respectively), and positively correlated with N:P and DIN (PC1 loadings of 0.64 and 0.49, respectively). The second PC axis explained 37.2% of the variability and was negatively correlated with all of the watershed chemistry variables (PC2 loadings of -0.45, -0.68, -0.52, and -0.25, for PO_4_^3−^, TSS, DIN, and N:P, respectively) (Table 2). The third and fourth PC axes explained 9.6% and 6.7% of the variability, respectively. Watershed identity and season were significantly related to the first two axes of the ordination space (r^2^ = 0.50 and 0.07, respectively; *p* = 0.001 in both cases).

**Table 2:** PCA axis loadings of dissolved inorganic nitrogen (DIN), phosphate (PO_4_^3−^), nitrogen:phosphorus ratio (N:P), and total suspended solids (TSS).

Parameter	PC1	PC2	PC3	PC4
DIN (mg/L)	0.49	− 0.52	0.39	− 0.58
PO_4_^3−^ (mg/L)	− 0.53	− 0.45	0.62	0.37
N:P ratio	0.64	− 0.25	− 0.07	0.72
TSS (mg/L)	− 0.26	− 0.68	− 0.68	− 0.07

**Fig. 5:**
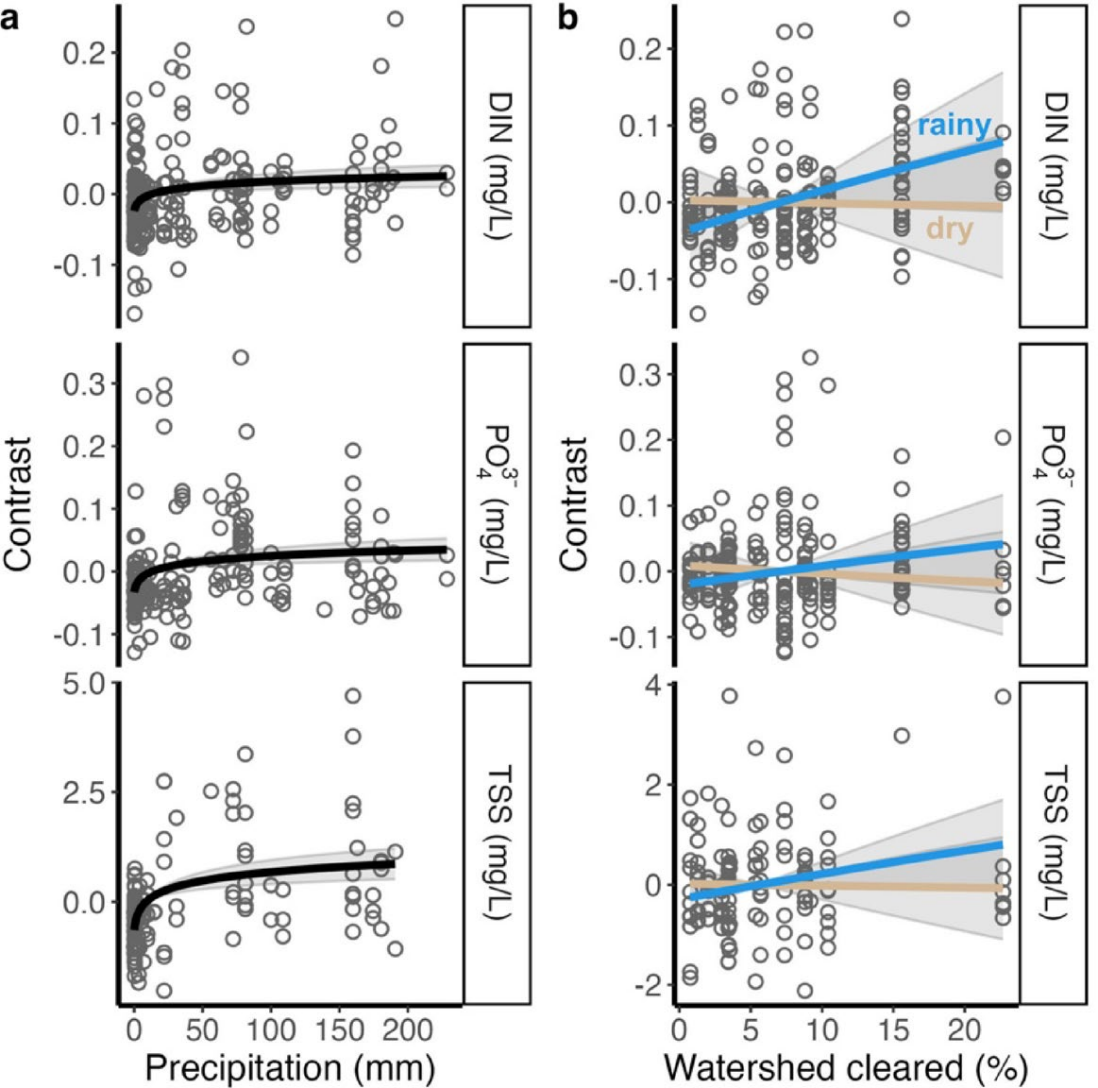
River chemistry in Moorea associated with patterns of rainfall and land use. Contrast plots reveal effects of recent precipitation (a) and seasonal role of land use (b; rainy season contrast shown in blue and dry season in tan) from mixed effects linear models of Dissolved Inorganic Nitrogen (DIN, top), phosphate (PO_4_^3−^, middle), and Total Suspended Solids (TSS, bottom).

Linear mixed effects models including population and precipitation, and cleared land percentage, season, and their interaction as fixed effects and watershed identity as a random effect revealed differential impacts of precipitation and cleared land on different components of surface water chemistry (Fig. 5, Supplementary materials S3). DIN was significantly positively associated with recent precipitation (*p* = 0.001), and a significant positive interaction between land clearing and season revealed that DIN was significantly higher under increased land clearing during the rainy season (*p* = 0.002). Similarly, PO_4_^3−^ was significantly higher following recent precipitation (*p* < 0.001), and higher under increased land clearing during the rainy season (*p* = 0.046). TSS was not significantly associated with land clearing, but was significantly higher following recent precipitation (*p* < 0.001).

### Water quality thresholds

Stringent and relaxed water quality thresholds were selected for both sediment and nutrient concentrations based on previous work in the tropics (5 mg/L and 50 mg/L, respectively, for TSS; and 0.1 mg/L and 0.18 mg/L, respectively, for DIN)^19,28,33,34^. Water samples from all watersheds, except for Ma’atea, exceeded the more stringent TSS water quality threshold of 5 mg/L at least once. On average, watersheds on Moorea exceeded this threshold in 39% of sampling events (Table 3). The maximum exceedance rate was found in Pihaena at 78% of the time. Eight of the watersheds included in this study exceeded the more relaxed TSS threshold of 50 mg/L at least once. Across Moorea, this threshold was exceeded on average 8% of the time. Six watersheds exceeded Hawaiian water quality standards with values of 50 mg/L at least 10% of the time (Haumi: 11%, Opunohu 1: 10%, Paopao 1 and 2: 14%, Papetoai: 12%, and Pihaena: 22%). All watersheds except Atiha and Vaianae exceeded the more stringent DIN thresholds at least once (Table 3). On average, stream samples across Moorea exceeded 0.1 mg/L DIN 47% of the time and 0.18 mg/L 21% of the time. Opunohu 2, Paopao 1, and Paopao 2 exceeded the more stringent DIN standard in every sample. Although the frequency by which rivers exceeded water quality thresholds was not significantly associated with the proportion of the watershed cleared of forest or population, the slope of the relationship between threshold exceedance frequencies and watershed clearing was almost always positive (i.e. β > 0, *p* > 0.05, except for the 0.18 mg/L DIN threshold).

**Table 3:** Exceedance percentages by watershed of low and high TSS and DIN water quality thresholds. Bold values represent exceedance frequencies of concern (> 10%).

Watershed	> 5 mg/L TSS	> 50 mg/L TSS	> 0.1 mg/L DIN	> 0.18 mg/L DIN
Atiha	8	0	0	0
Ha’apiti	**50**	8	**24**	0
Haumi	**22**	**11**	**78**	**44**
Ma’atea	0	0	**11**	0
Maharepa	**42**	0	**12**	6
Opunohu 1	**50**	**10**	7	0
Opunohu 2	**30**	0	**100**	**77**
Paopao 1	**43**	**14**	**100**	**71**
Paopao 2	**43**	**14**	**100**	**14**
Papetoai	**62**	**12**	**88**	**25**
Pihaena	**78**	**22**	**23**	**13**
Teavaro	**50**	0	**73**	**27**
Vaianae	**33**	8	0	0
Overall	**39**	8	**47**	**21**

## Discussion

Through a combination of island-wide water sampling and remote sensing analyses, we identified associations between land use at the watershed scale and the nutrient and sediment concentrations of rivers on Moorea. Dissolved inorganic nitrogen (DIN), phosphate (PO_4_^3−^), total suspended solids (TSS), and the nitrogen to phosphorus (N:P) ratio differed across rivers and between the rainy and dry seasons. Further, DIN and PO_4_^3−^ concentrations were positively related to the percentage of land in a watershed that was cleared of forest during the rainy season, and DIN, PO_4_^3−^, and TSS were all higher following recent precipitation. Importantly, DIN and TSS concentrations regularly exceeded thresholds established in similar systems to identify watersheds that may pose a danger to human health and that of aquatic biota.

### Nutrient enrichment and its implications

In the linear models, we observed a positive relationship between land-clearing and high DIN concentrations during the rainy season, and generally higher DIN following recent precipitation. Similar relationships between DIN and land-clearing combined with human population were found in studies of rivers in American Samoa^33^ and Guam^34^. Although we did not identify significant associations between DIN and population on Moorea, the delineation of census zones on Moorea does not perfectly correspond with the watershed boundaries in our study. Our results indicate that watershed cleared land may be sufficient to make approximate predictions of river DIN concentrations in steep tropical islands. This could be a valuable tool to remotely approximate DIN concentrations in unstudied watersheds on steep-islands, using only satellite-derived data on cleared land. It is important to note that a more complex runoff model would be required to calculate flux of DIN from watersheds^35^. However, estimates of DIN concentrations can be valuable in assessing potential human or environmental health concerns^33,34^ and regulatory thresholds are commonly based on concentrations rather than fluxes^27^.

We observed higher DIN concentrations in rivers on Moorea than previous work on tropical islands. The highest observed DIN sample in our study, 0.53 mg/L (37.72 µM), was more than double the maximum measured in Guam (0.20 mg/L)^33^, but lower than the maximum values found in American Samoa (0.75 mg/L)^34^. Another study in American Samoa recorded a mean of 8.5 µM DIN in a heavily disturbed watershed^25^ which is close to the overall mean observed in our study (0.14 mg/L, or 10.0 µM), including data from all watersheds spanning minimally to highly disturbed. As a result of this difference, DIN concentrations in almost all watersheds on Moorea exceeded the water quality threshold of 0.1 mg/L established in American Samoa. DIN values in Moorea also frequently exceeded the more relaxed 0.18 mg/L threshold from the Hawaii water quality standards, despite being comparable to watersheds in Hawaii with similar land use^36,37^.

Our linear mixed models indicate that DIN enrichment in streams of Moorea is primarily a result of surface runoff, given its positive association with recent precipitation and with cleared land during the rainy season. The percent cleared land classification in our satellite image analysis is largely comprised of agricultural land; one of the primary commercial crops grown on Moorea is pineapples^20^. Pineapple farming requires year-round applications of N fertilizer and has contributed to N-loading in rivers across the tropics^38–40^. The N in these fertilizers is usually in the form of urea which transforms to NH_4_^+^ and then NO_3_^−^ via ammonification and nitrification in the soil due to biological activity^41^. NO_3_^−^ was the most abundant form of N we observed. NO_3_^−^ is highly water soluble and thus is easily transported from terrestrial to aquatic environments via surface runoff. N-enriched runoff is likely exacerbated on Moorea by the lack of runoff control and the lack of requirements to keep riparian buffers between fields and rivers^42^.

Previous work on Moorea measuring N isotopes in nearshore macroalgae observed elevated δ15N in algae tissue sampled near population centers indicating that sewage leaching directly into the nearshore environment via submarine groundwater discharge was likely a large contributor to N pollution^31,43,44^. Direct measurements of DIN concentrations in groundwater and submarine groundwater seeps in these studies recorded DIN levels as high as 45 µM^44^. Such groundwater seeps could be at the root of high DIN concentrations reported in American Samoa, described above. It is likely that groundwater carrying DIN is also entering streams through hyporheic exchange in the lower reaches of the watershed due to the porous nature of the volcanic geology^45^ and high head pressure from the island’s short, steep slopes^46^. This exchange may be enhanced in the lower reaches of the rivers as they have been heavily channelized and fortified for flood control (*KN pers. obs.*) which has enhanced stream bed erosion and deepening of river channels^16^ resulting in an increase in head near the terminus where our samples were collected.

The range of PO_4_^3−^ concentrations recorded in this study are comparable to those found in a study of watersheds on Oahu, Hawaii, across base and stormflow conditions, and including watersheds considered to be forested, agricultural and urban^36^. In our linear mixed effects models, riverine PO_4_^3−^ on Moorea was only weakly associated with land clearing during the rainy season and not associated with population, suggesting that agriculture and urbanization were not directly responsible for the differences in PO_4_^3−^ concentrations we observed around the island. Anthropogenic sources of PO_4_^3−^ often come in the form of agricultural fertilizers, however pineapples need little to no P fertilizer where soils have a naturally high P content as they likely do on Moorea^47^. Soils on the island are derived from erosion of volcanic basalts which tend to be rich in P, and the availability of P in the soil of a given area is additionally dependent on numerous factors including age, weathering, and localized precipitation^48^. PO_4_^3−^ concentrations in rivers are often tied to sediment concentrations as PO_4_^3−^ can desorb from sediment particles in transit^49^; this pattern was evident in our dataset as well. As there are at least eight different volcanic soils on Moorea^50 ^differences in riverine PO_4_^3−^ among rivers could be related to erosion, vegetative utilization, or leaching of soils with different PO_4_^3−^ levels, depending on the predominant surface soil types in a given watershed. Groundwater and submarine groundwater discharge on Moorea is also elevated in PO_4_^3−^ relative to surface water, probably due to weathering of PO_4_^3−^ rich basaltic rock in aquifers^44^. It is also likely that PO_4_^3−^ also enters streams on Moorea via hyporheic exchange. As anthropogenic sources of DIN increase on the island due to expanded agriculture and urbanization, it is likely that the N:P ratio will also increase as N enrichment outpaces PO_4_^3−^ mobilization.

### Drivers and consequences of patterns in riverine suspended solids

The linear regression analysis illustrated that TSS concentrations were strongly positively related to recent precipitation, indicating that climatic drivers play an important role in the mobilization of sediment. Similar statistical tools have been used to predict TSS in other mountainous watersheds^51^. These models do not predict discharge or sediment flux, but like our DIN model they could be a useful framework for a first order assessment of potential human health or environmental impact concerns in similar regions. TSS thresholds and regulations are often based on concentrations, not flux^27^. In locations where data are lacking, such as many high islands in the South Pacific, this simple linear modeling approach may provide valuable insights where there is insufficient data to inform a more complicated runoff model like the Soil and Water Assessment Tool (SWAT), Revised Universal Soil Loss Equation (RUSLE), Modified Morgan–Morgan–Finney (MMF) model, or others that are commonly paired with remote sensing analysis to assess relationships between land use and TSS^52–54^.

TSS concentrations in all studied watersheds on Moorea, except for Ma’atea, exceeded the water quality threshold of 5 mg/L TSS for safe drinking, bathing and cleaning^28,38^. TSS values from eight watersheds also exceeded the 50 mg/L threshold representing potentially lethal levels for fish species^28 ^for an overall exceedance rate of 8%. We also observed high variability in TSS levels between samples, including some outliers (e.g. >900 mg/L compared to mean of 137 mg/L in Paopao 1) that illustrate how sediment is mobilized in these flashy systems and how difficult it can be to capture differences between watersheds or seasons when much of the sediment that moves from land to river can mobilize during a few major rain events^55^. The range of TSS values observed across watersheds experiencing varying degrees of land clearing and urbanization are within the ranges observed in a study across an urbanization gradient in Hawaii^56^ and a study of a “pristine” rainforest watershed in Fiji that includes samples taken during a cyclone^57^. TSS values observed in the most disturbed watersheds of our study (Paopao and Opunohu) were considerably lower than those observed in a study of urban and developing watersheds on Tahiti which recorded values as high as 33.3 g/L TSS in a watershed that was actively undergoing sizeable earthworks projects^58^.

Much of the existing land suitable for development in Moorea has been utilized, so new residential or industrial development requires considerable earth moving efforts, including forest clearing and terracing to create flat buildable plots. We did not observe use of soil retention measures (e.g. vegetative cover, mulch, silt fences, geotextiles) that have been used in similar steep tropical systems to reduce erosion^59,60^. Furthermore, a majority of the roads in the interior of the island are not paved, tend to follow river channels, and lack any soil retention devices (*KN pers. obs.)* that have been successfully demonstrated in comparable systems^61^. As a result of their demonstrated ability to reduce erosion, implementation of erosion control measures such as those listed above are required by law on construction projects in Hawaii^27^.

Although we did not detect a statistically significant effect of land clearing, the positive slope between TSS and clearing during the rainy season matched our expectation that agriculture is plays a role in TSS runoff, given that pineapple farming is known to contribute to high erosion rates^62^. In a recent study which scored farming practices in French Polynesia based on soil preservation, pineapple farming scored the lowest for its contributions to soil degradation^20^. Across French Polynesia and in many other tropical climates pineapples are often grown on moderate to steep slopes making it difficult to employ erosion control measures such as cover crop or mulching between rows, which have been shown to be effective in reducing soil erosion in other agricultural systems^20,38^. Further, to maximize yield from a farmed plot, many farms in Moorea extend right up to riverbanks with no riparian zone or other buffer to slow the movement of sediment from the fields to the rivers (Neumann *pers. obs.*). All of these conditions create high erosion potential, and contribute to the strong relationship between land clearing and TSS in rivers we observed in Moorea.

## Conclusion

This study provides the first island-wide assessment of riverine nutrient and sediment concentrations on Moorea, French Polynesia, and explores the relationships between river chemistry, weather, land use, and populations. Watersheds on Moorea, as well as other small high tropical islands worldwide, are developing rapidly with growing populations, tourism, logging and large-scale agriculture^11,63^. Our study puts Moorea in context of these other efforts to study land use and its impacts on rivers. The relationships we observed between land use and river nutrient chemistry on Moorea are comparable to those observed in other developed and developing islands around the world^19,25,26^. In some cases, Moorea may be more susceptible to unsafe freshwater conditions as indicated by our comparison with other studies with respect to TSS and DIN threshold exceedance. Perhaps most urgently, this study highlights watersheds on Moorea which present risks for human health and/or concerns regarding ecosystem processes based on nutrient and sediment thresholds. Finally, this study also serves as a point of comparison against future changes on Moorea as land clearing and population growth continue on the island. As Moorea, and other high tropical islands, continue to develop, impacts of watershed change are likely to increase with potentially profound consequences for human and ecosystem health.

## Methods

### Study location

Moorea is a volcanic island located in the Society Islands Archipelago of French Polynesia (17° 29′ S, 149° 50′ W). As the island eroded and subsided, a barrier reef formed separating the open ocean from a 53 km^2^ lagoon^64^. The island has a relatively small area of 134 km^2^, but features diverse topography. Since it was formed by a volcanic eruption approximately 1.6 million years ago^65 ^Moorea has collapsed and eroded into numerous distinct watersheds separated by steep, sharp ridgelines with a highest elevation of 1207 m at Mount Tohiea^65^ (Fig. 1). A majority of the soils (98.7%) on the island formed from weathering and erosion of volcanic parent material with the remainder being coralline in origin^50^. Of the eight soils of volcanic origin on Moorea, five originate from weathered basalt^50^.

Moorea has been inhabited by humans since around 200 CE when it was settled by the Polynesians^66 ^who brought with them a variety of nonnative plants and animals for cultivation. A 2015 study of vegetation on Moorea classified 32% of the island as urbanized and cultivated lands, 17% as novel habitat dominated by invasive species, 45% as hybrid habitats featuring a mix of introduced and native species, and 6% as native habitat which is primarily located on extremely steep slopes and at high elevation^65^. As of the 2017 census, Moorea was home to 17,357 permanent residents^67^.

The largest watersheds on Moorea are two adjacent valleys on the north shore, Opunohu and Paopao (Table 1), formed by a collapse of the volcano’s caldera^65^. Opunohu is home to a small population (159 people as of 2017), but hosts a large portion of the island’s commercial agriculture which is principally comprised of pineapple plantations and cattle pasture. Using the Strahler method, the Opunohu River is one of the only 4th order streams on the island^68^. As an example of the steepness of watersheds on Moorea, more than 90% of the Opunohu valley is comprised of slopes greater than 10%^69^. Neighboring Paopao is home to the largest population center (1281 people as of 2017) and small mixed agricultural fields, and also features a 4th order stream. Its predominant sub-watersheds feature 2nd-4th order streams and span a range of areas. The smallest standalone watershed included in this study, Teavaro, is located on the eastern shore of the island and is only 1.3 km^2^, and home to 367 people. Teavaro and the rest of the rivers in this study are second and third order streams. The variety of watersheds in close proximity featuring a range of areas, land uses and populations makes Moorea a compelling location to investigate interactions between human activity and riverine nutrients and sediments.

In order to inform our study of rivers and land use on Moorea, we first engaged the local community to receive feedback regarding key questions that would be of interest and value to the people of Moorea. The goals, objectives, and research locations for this study were shaped by meetings with community members organized by the Te Pū ‘Ātiti’a Cultural Center located directly adjacent to the University of California Gump Research Station, where our research was based. These conversations precipitated a new community science collaborative project on Moorea called ‘ātivai. The name “‘ātivai” is a merging of the words ‘ātiti’a (the clan who stands) and “vai” (fresh water) into ‘ātivai (the water clan) to represent the collaboration of ‘ātiti’a community members and fresh water scientists in this project. Members of ‘ātivai contributed to the study design, provided local knowledge and site context, and aided with field data collection throughout this study. The combination of modern scientific tools and techniques with traditional and local knowledge of the island and its rivers was paramount to the success of this research.

### Focal watershed selection

In the first year, eight watersheds were chosen to represent a range of watershed sizes, land uses and population densities, and are located on all three shores of the island. These watersheds were: Atiha, Ha’apiti, Maharepa, Opunohu, Paopao, Pihaena, Teavaro, and Vaianae. The Opunohu watershed was evaluated at the level of its two primary sub-watersheds, which have vastly different human populations (Fig. 1; Table 1). Three additional watersheds - Haumi, Ma’atea, and Papetoai – as well as the primary sub-watersheds of the Paopao watershed (defined by 2nd − 4th order streams), were added in the second year of sampling to increase sampling across the ranges of watershed size and human impacts (Fig. 1; Table 1). All of the rivers included in this study are perennial, with the exception of Pihaena which has low or no flow during the dry season.

### Water sample collection

We collected water samples during two rainy and two dry seasons between January 2018 and September 2019 (rainy: January - March 2018 and February - March 2019, dry: August - September 2018 and 2019). During the study periods, water samples were collected as close to the mouths of the rivers as possible, but above brackish mixing zones. Where natural or manmade impediments to access existed, we made every effort to find a suitable sampling location as close to the river mouth as possible. During the above periods, samples were collected at least once a week with attention paid to capturing samples across river stages from low-flow to storm-flow. We collected storm-flow samples during or shortly after rain events to ensure that we captured the nutrient and sediment regimes during times of high flow. In total, 228 samples were collected over the four sampling seasons (37 and 56 samples in the 2018 dry season and rainy season, respectively, and 53 and 82 samples in the 2019 dry season and rainy season, respectively).

River samples were collected at approximately 60% water depth (e.g. at 40 cm above the bottom in a 1 m deep river) at the center of flow. Samples were collected in acid washed 1-liter HDPE Cubitainers^®^, transported on ice to the lab at Gump Station, and kept refrigerated until processed. Samples were processed upon returning to the lab within no more than 24 h. Each sample was divided and filtered for nutrients or total suspended solids using a multi-channel peristaltic filtration system.

### Nutrient analyses

Nutrient sub-samples were filtered through 47 mm 0.15 μm PES filter discs (Sterlitech ©) and then divided for separate analytical methods. NH_4_^+^ analyses were conducted immediately after filtration using the OPA method^70^ on a Turner Trilogy fluorometer fitted with an ammonium detection module (Minimum Detection Limit (MDL): 0.90 mg/L). The remaining samples were frozen at -20 °C and transported to the Centre de Recherches Insulaires et Observatoire de l’Environnement (CRIOBE) on Moorea where they were analyzed for PO_4_^3−^ (MDL: 0.28 mg/L), NO_3_^−^ (MDL: 0.12 mg/L), and NO_2_^−^ (MDL: 0.092 mg/L) on an AA3 Auto-analyzer (SEAL Analytical, e.g^71^. Total DIN per sample represents the molar sum of NO_2_^−^, NO_3_^−^, and NH_4_^+^, and was converted to mg/L of N only for comparison with other reported values. The molar N:P ratio was calculated using the sum of the molarities of NO_3_^−^, NO_2_^−^, and NH_4_^+^ (i.e. molarity of DIN) divided by the molarity of PO_4_^3−^.

### Total suspended solids (TSS) analysis

TSS samples were filtered using pre-dried and pre-weighed 47 mm GF/F (0.70 μm) filter discs (Whatman^®^). Filters were weighed on a Mettler-Toledo XS104 balance with 0.1 mg precision. Samples were filtered to 200 mL, or until the filters were completely clogged preventing more water to pass through, whichever came first. If filters clogged before filtering 200mL, we recorded the total volume filtered. After filtration, the GF/F filters were placed in a drying oven at 95 °C for a minimum of 24 h at which time they were re-weighed. TSS was calculated using Eq. 1:

1 $$\:TSS=\:\frac{\left(post{\text{-}}filtering\:and\:drying\:weight\right)-(pre{\text{-}}filtering\:weight)}{volume\:filtered}$$

### Land cover classification

The Worldview-3 (WV3) Satellite (Maxar Technologies) collects repeat imagery with the 8-band WV110 sensor every 4.5 days with a ground sampling distance of 1.38 m. We used a series of WV3 images collected during four days in 2018 that had little-to-no cloud cover. The imagery was aligned, georeferenced, orthorectified, and mosaicked using the Ortho Mapping workspace in ArcGIS Pro v3.1.2. Training data for land cover classification were generated using data from the Direction des Affaires Foncières de la Polynésie Française (the Directorate of Land Affairs, French Polynesia), and included the classes Water, Forest, Monoculture (intensive agriculture), Buffer/Agriculture (permaculture, orchards, and other vegetated, non-forested land), Buildings, Paved, Dirt and Sand. A U-Net pixel classifier was trained on a ResNet-50 architecture with 10% of training samples withheld for validation for each of the unique dates of imagery collection, yielding an accuracy of 0.86 ± 0.04 (mean ± std. dev. across date-wise models). Individual layers were then compiled into a “consensus” land cover map that used the modal classification for each pixel (Fig. 1, [ref^72^]). This approach reduced noise related to solar and topographic variability in pixel classification outputs from individual date layers, and overcame gaps in the dataset introduced by cloud cover. The consensus land cover map had a 98% island-wide classification accuracy.

Watersheds were delineated using the Hydrology tool in the Spatial analyst toolbox in ArcGIS Pro v3.1.2. Using a digital elevation model (DEM) from the Shuttle Radar Topography Mission Version 3 (30 m resolution)^73^, we identified the watersheds that feed into our river sampling locations using the Flow Accumulation and Flow Direction Tools in ArcGIS. Points along the rivers were identified towards the mouth of rivers of interest using the Snap Pour Point tool. Watersheds of interest for the project were then delineated using the Watershed tool.

### Precipitation and census data

Daily precipitation data for Moorea for 2018 and 2019 were obtained for 5 meterological stations on the island through a license agreement with Meteo France. Census data for 2017 was obtained via a license agreement between the Gump Research Station and the Institut de Statistiques de la Polynésie Française. The census data were originally collected in 102 districts on the island, often with multiple districts located inside of a watershed. In order to determine the population for each watershed, we aggregated census data based on spatial intersections between census districts and focal watersheds as described above.

### TSS and DIN concentration thresholds

Low (more relaxed) and high (more stringent) water quality thresholds for both DIN and TSS concentrations were selected from a review of literature from similar systems including Hawaii, Guam, the Solomon Islands and American Samoa. The low TSS threshold (5 mg/L) represents concerns for human consumption and bathing^19,28^. The high TSS threshold (50 mg/L) is based on water quality standards established in Hawaii^27^ and is based on the levels at which exposure to suspended sediment become lethal to a majority of fish species^28^. According to the Hawaiian standards, TSS in streams should not exceed 50 mg/L for more than 10% of the time in the rainy season. The lower DIN concentration threshold of 0.1 mg/L was selected based on studies of runoff impacts to nearshore reefs in American Samoa^33^ and Guam^34^. An exceedance of this threshold in streams for more than 20% of the time was associated with diminished diversity and size of coral communities in both regions. The higher threshold of 0.18 mg/L DIN was based on Hawaiian water quality standards which state that streams should not exceed this threshold more than 10% of the time in the rainy season^27^. We calculated the percentage of time that samples collected from each watershed on Moorea exceeded these thresholds using Eq. 2:

2 $$\:\%\:exceedance=\:\frac{\#\:samples\:from\:watershed\:that\:exceed\:threshold}{total\:\#\:samples\:from\:watershed}*100$$

### Statistical analyses

We used linear models to analyze differences in river chemistry (DIN, NO_3_^−^, NO_2_^−^, NH_4_^+^, PO_4_^3−^, or TSS; the response variable of each model) by watershed, season, and their interaction. To investigate differences between sites, we used post-hoc Tukey tests with adjusted alpha (p-value) based on the studentized range statistic. Data were 1 + log transformed prior to analysis to improve model fit, based on inspection of q-q plots and residuals using the ‘qqplot’, ’qqnorm’, and ‘resid’ functions in R v4.2.2^74^. Only watersheds that had samples from the wet and dry seasons were included in these models, so Pihaena was excluded because it only flowed in the wet season.

To assess differences in river chemistry among sampling locations during both seasons, we used a principal component analysis (PCA). PCA is a form of ordination analysis based on Euclidean distance that is well suited to complex datasets with covarying variables. Differences across watersheds and seasons were assessed based on a correlation matrix of DIN, PO_4_^3−^, N:P, and TSS levels at a given sampling event. We used the ‘envfit’ function in the R package ‘vegan’ to test whether watershed identity or season explained variation in river chemistry^75^. We evaluated direct associations between river chemistry and environmental variables using a series of linear mixed effects models testing how watershed factors predict nutrient and sediment concentrations. We included river chemistry measurements (DIN, PO_4_^3−^, and TSS) as response variables; population and precipitation, as well as the percent of watershed area cleared, season, and their interaction as fixed predictors; and watershed identity as a random intercept. All environmental data and river chemistry data were 1 + log transformed before analysis because they were not normally distributed. Multicollinearity was assessed using variance inflation factor (VIF); all environmental variables had VIF values < 5 and were taken to be acceptable.

## Supplementary Information

Below is the link to the electronic supplementary material.

Supplementary Material 1

## Data Availability

Data from this study are stored on EDI (https://doi.org/10.6073/pasta/a882a1478cdf99da4473449024f26229), and code to reproduce analyses are on github (https://github.com/JepsonNomad/MooreaRiverChemistry).
