# Supplementary material for: Land use shapes riverine nutrient and sediment concentrations on Moorea, French Polynesia
Source: Sci Rep. 2025 Jul 31;15:27948. doi: 10.1038/s41598-025-13425-1 (PMC12314069; doi:10.1038/s41598-025-13425-1)
Supplement: Supplementary file 1 — Supplementary Material 1 [file 41598_2025_13425_MOESM1_ESM.pdf]

Supplementary materials
Neumann et al. revised for *Scientific Reports*
Land use shapes riverine nutrient and sediment concentrations on Moorea, French Polynesia

Supplementary materials S1. Water chemistry variability in space and time
Mean, range, and standard deviation of Dissolved Inorganic Nitrogen, Phosphate, N:P Ratio, and Total Suspended Solids in each watershed.

	Dissolved Inorganic Nitrogen (DIN; mg/L)			*Phosphate (PO_4^{3-}; mg/L)*			*N:P ratio*			*Total Suspended Solids (TSS; mg/L)*		
Site	***mean***	***range***	***SD***	***mean***	***range***	***SD***	***mean***	***range***	***SD***	***mean***	***range***	***SD***
Atiha	0.06	0.02-0.09	0.02	0.15	0.08-0.27	0.05	2.72	0.93-4.51	1.11	5.39	0-38.12	10.4
Ha'apiti	0.08	0.02-0.13	0.03	0.11	0.07-0.24	0.05	5.4	1.91-8.22	2.16	14.23	0-81.25	24.01
Haumi	0.18	0.08-0.28	0.08	0.15	0.09-0.3	0.06	8.48	4.49-13.17	3.55	12.26	1.25-81.88	26.2
Ma'atea	0.07	0.01-0.12	0.04	0.11	0.08-0.15	0.03	4.2	0.68-7.53	2.13	2.53	0.5-4	1.31
Maharepa	0.07	0.01-0.38	0.09	0.21	0.15-0.32	0.05	2.29	0.51-11.38	2.65	7.71	0-20.62	7.47
Opunohu												
Opunohu 1	0.06	0.02-0.12	0.02	0.12	0.05-0.48	0.11	5.03	0.29-10.27	2.6	11.09	1.25-72.5	21.65
Opunohu 2	0.25	0.12-0.51	0.12	0.11	0.06-0.28	0.06	19.74	2.79-48.58	13.75	7.54	0.62-40	11.67
Paopao	0.26	0.09-0.53	0.1	0.11	0.03-0.26	0.06	18.7	6.59-39.36	8.99			
Paopao 1	0.23	0.14-0.43	0.09	0.09	0.02-0.19	0.06	24.17	8.15-54.7	15.8	136.95	1-901.67	337.35
Paopao 2	0.17	0.14-0.24	0.03	0.17	0.05-0.45	0.14	10.64	2.27-20.98	7.25	117.19	1.5-783.33	293.78
Paopao 3	0.15	0.07-0.32	0.09	0.26	0.08-0.72	0.18	4.68	1.47-6.79	2.06			
Papetoai	0.14	0.02-0.27	0.07	0.22	0.15-0.28	0.05	4.35	0.79-9.13	2.57	45.86	2-293.75	100.69
Pihaena	0.08	0-0.35	0.09	0.29	0.12-0.69	0.16	1.92	0.04-5.14	1.55	29.58	2.5-126.25	41
Teavaro	0.16	0.01-0.33	0.11	0.13	0.02-0.23	0.05	8.19	2.77-17.27	4.73	5.55	1-12.5	3.63
Vaianae	0.06	0.02-0.09	0.02	0.1	0.05-0.27	0.05	4.47	1.41-6.29	1.44	8.24	1-58.75	16.04

Supplementary materials
Neumann et al. revised for *Scientific Reports*
Land use shapes riverine nutrient and sediment concentrations on Moorea, French Polynesia

Supplementary materials S2. Results from ANOVA of river chemistry
ANOVA summaries for linear models of each major water chemistry parameter as a function of season, watershed (site), and their interaction. Water chemistry parameters as follows: Dissolved Inorganic Nitrogen (DIN), Nitrite ($NO2^-$), Nitrate ($NO3^-$), Ammonium ($NH4^+$), Phosphate (PO_4^{3-}), and Total Suspended Solids (TSS).

Response	Term	Df	Sum Sq	Mean Sq	F value	p
**DIN**	*Season*	1	0.01	0.01	4.13	0.044*
	Site	13	0.81	0.06	18.77	0***
	Season:Site	13	0.11	0.01	2.57	0.003**
	Residuals	156	0.52	0		
**NO_2^-**	*Season*	1	0	0	1.77	0.185
	Site	13	0.02	0	16.57	0***
	Season:Site	13	0	0	2.76	0.001***
	Residuals	156	0.01	0		
**NO_3^-**	*Season*	1	0.13	0.13	5.21	0.024*
	Site	13	7.2	0.55	21.84	0***
	Season:Site	13	1	0.08	3.04	0.001***
	Residuals	156	3.96	0.03		
**NH_4^+**	*Season*	1	0	0	0	0.961
	Site	13	0.01	0	5.81	0***
	Season:Site	13	0	0	1.15	0.318
	Residuals	163	0.02	0		
**PO_4^{3-}**	*Season*	1	0.02	0.02	5.05	0.026*
	Site	13	0.28	0.02	6.25	0***
	Season:Site	13	0.08	0.01	1.77	0.053.
	Residuals	156	0.54	0		
**TSS**	*Season*	1	12.53	12.53	10.49	0.002**
	Site	11	19.75	1.8	1.5	0.144
	Season:Site	11	17.18	1.56	1.31	0.233
	Residuals	93	111.1	1.19		

Supplementary materials S3. Results from mixed-effects models of river chemistry

Linear mixed effects model results comparing water DIN, PO_4^{3-}, and TSS with log-transformed population, precipitation and cleared area, season, and their interaction as fixed effects, and site identity as a random effect.

Total Suspended Solids (TSS):

	log TSS		
Predictors	*Estimates*	*CI*	*p*
(Intercept)	0.59	-0.66 – 1.84	0.349
logCleared	-0.44	-7.32 – 6.43	0.898
Season [r]	-0.15	-0.77 – 0.47	0.630
logPopulation	0.11	-0.10 – 0.32	0.290
logPrecipitation	0.29	0.17 – 0.41	**<0.001**
logCleared × Season [r]	5.78	-2.32 – 13.88	0.160
**Random Effects**			
σ^2	1.08		
$\tau_{00\ Site}$	0.08		
ICC	0.07		
N_{Site}	15		
Observations	127		
Marginal R^2 / Conditional R^2	0.273 / 0.322		

Dissolved Inorganic Nitrogen (DIN):

	log DIN		
Predictors	*Estimates*	*CI*	*p*
(Intercept)	0.15	-0.02 – 0.31	0.078
logCleared	-0.04	-0.73 – 0.65	0.905
Season [r]	-0.07	-0.10 – -0.04	**<0.001**
logPopulation	-0.01	-0.03 – 0.02	0.717
logPrecipitation	0.01	0.00 – 0.01	**0.001**
logCleared × Season [r]	0.62	0.24 – 1.00	**0.002**
**Random Effects**			
σ^2	0.00		
$\tau_{00\ Site}$	0.00		
ICC	0.51		
N_{Site}	15		
Observations	213		
Marginal R^2 / Conditional R^2	0.083 / 0.548		

Phosphate (PO_4^{3-}):

	log PO_4^{3-}		
Predictors	*Estimates*	*CI*	*p*
(Intercept)	0.13	-0.00 – 0.26	0.054
logCleared	-0.13	-0.71 – 0.45	0.653
Season [r]	-0.03	-0.06 – 0.01	0.150
logPopulation	-0.00	-0.02 – 0.02	0.858
logPrecipitation	0.01	0.01 – 0.02	**<0.001**
logCleared × Season [r]	0.44	0.01 – 0.87	**0.046**
**Random Effects**			
σ^2	0.00		
$\tau_{00\ Site}$	0.00		
ICC	0.32		
N_{Site}	15		
Observations	213		
Marginal R^2 / Conditional R^2	0.101 / 0.386		
